# Evaluation of the Relationships between Simple Anthropometric Measures and Bioelectrical Impedance Assessment Variables with Multivariate Linear Regression Models to Estimate Body Composition and Fat Distribution in Adults: Preliminary Results

**DOI:** 10.3390/biology10111209

**Published:** 2021-11-19

**Authors:** Danúbia da Cunha de Sá-Caputo, Anelise Sonza, Ana Carolina Coelho-Oliveira, Juliana Pessanha-Freitas, Aline Silva Reis, Arlete Francisca-Santos, Elzi Martins dos Anjos, Laisa Liane Paineiras-Domingos, Thais de Rezende Bessa Guerra, Amanda da Silva Franco, Vinicius Layter Xavier, Claudia Jakelline Barbosa e Silva, Marcia Cristina Moura-Fernandes, Vanessa Amaral Mendonça, Ana Cristina Rodrigues Lacerda, Alessandra da Rocha Pinheiro Mulder, Aderito Seixas, Alessandro Sartorio, Redha Taiar, Mario Bernardo-Filho

**Affiliations:** 1 Programa de Pós-Graduação em Ciências Médicas, Faculdade de Ciências Médicas, Universidade do Estado do Rio de Janeiro, Rio de Janeiro 20511-010, Brazil; dradanubia@gmail.com (D.d.C.d.S.-C.); fisio.alinereis@hotmail.com (A.S.R.); laisanit@gmail.com (L.L.P.-D.); marciafernandesfisio@hotmail.com (M.C.M.-F.); 2 Programa de Pós-Graduação em Fisiopatologia Clínica e Experimental, Faculdade de Ciências Médicas, Universidade do Estado do Rio de Janeiro, Rio de Janeiro 20511-010, Brazil; 3 Laboratório de Vibrações Mecânicas e Práticas Integrativas—LAVIMPI, Departamento de Biofísica e Biometria, Instituto de Biologia Roberto Alcântara Gomes and Policlínica Piquet Carneiro, Universidade do Estado do Rio de Janeiro, Rio de Janeiro 20950-003, Brazil; ju.freitas.fisio@gmail.com (J.P.-F.); elzimartins45@gmail.com (E.M.d.A.); bernardofilhom@gmail.com (M.B.-F.); 4 Departamento de Fisioterapia, Faculdade Bezerra de Araújo, Rio de Janeiro 23052-180, Brazil; fisioarlete@hotmail.com; 5 Mestrado Profissional em Saúde, Medicina Laboratorial e Tecnologia Forense, Universidade do Estado do Rio de Janeiro, Rio de Janeiro 20950-003, Brazil; 6 Programa de Pós-Graduação em Fisioterapia e Programa de Pós-Graduação em Ciências do Movimento Humano, Departamento de Fisioterapia, Universidade do Estado de Santa Catarina—UDESC, Florianópolis 88035-901, Brazil; anelise.sonza@udesc.br; 7 Departamento de Fisioterapia, Instituto de Ciências da Saúde, Universidade Federal da Bahia, Salvador 40231-300, Brazil; 8 Instituto de Nutrição do Cérebro e Coração, Itaipú, Niterói, Rio de Janeiro 24342-440, Brazil; thaisbessa@id.uff.br; 9 Departamento de Nutrição, Faculdade Bezerra de Araújo, Rio de Janeiro 23052-180, Brazil; franco.amandarj@gmail.com; 10 Coordenação de Nutrição, Centro de Ciências da Saúde, Centro Universitário Serra dos Órgãos, Teresópolis 25964-000, Brazil; 11 Secretaria de Saúde, Prefeitura Municipal de Duque de Caxias, Duque de Caxias, Rio de Janeiro 25070-005, Brazil; 12 Departamento de Estatística, Instituto de Matemática e Estatística, Universidade do Rio de Janeiro, Rio de Janeiro 20550-900, Brazil; viniciuslx@ime.uerj.br; 13 Programa de Pós-Graduação em Ciências Computacionais, Instituto de Matemática e Estatística, Universidade do Rio de Janeiro, Rio de Janeiro 20550-900, Brazil; claudia.jakelline@gmail.com; 14 Faculdade de Ciências Biológicas e da Saúde, Universidade Federal dos Vales do Jequitinhonha e Mucuri (UFVJM), Diamantina 39100-000, Brazil; vaafisio@hotmail.com (V.A.M.); lacerdaacr@gmail.com (A.C.R.L.); 15 Departamento de Nutrição Aplicada, Universidade do Estado do Rio de Janeiro, Rio de Janeiro 20511-010, Brazil; alessandra.mulder@gmail.com; 16 Escola Superior de Saúde Fernando Pessoa, Fundação Fernando Pessoa, 4200-253 Porto, Portugal; aderito@ufp.edu.pt; 17 Experimental Laboratory for Auxo-Endocrinological Research, Istituto Auxologico Italiano, IRCCS, 20145 Milan & Division of Metabolic Diseases & Auxology, 28824 Verbania, Italy; sartorio@auxologico.it; 18 MATIM, Moulin de la Housse, Université de Reims Champagne Ardenne, CEDEX 02, 51687 Reims, France; redha.taiar@univ-reims.fr

**Keywords:** body composition, fat distribution, bioelectrical impedance, anthropometry

## Abstract

**Simple Summary:**

Overweight and obesity are associated with accumulation of abdominal fat, increasing chronic diseases, cardiovascular risk and mortality. Although the evaluation of body composition and fat distribution are highly relevant, the high cost of the gold standard techniques limits their wide utilization. Therefore, the aim of this work was to explore the relationships between simple anthropometric measures and bioelectrical impedance analyzes (BIA) variables using multivariate linear regression models to estimate body composition and fat distribution in adults. In this cross-sectional study, sixty-eight adult individuals performed BIA, anthropometric measurements [waist circumference (WC), neck circumference (NC), mid-arm circumference (MAC)], conicity index (C-index), fat mass/fat-free mass (FM/FFM) ratios, body mass index (BMI) and body shape index (ABSI). Statistical analyzes were performed with the R program, considering *p* ≤ 0.05 as significant. BIA variables with the highest correlations with anthropometric measures were total body water (TBW), body fat percentage (BFP), FM, FFM and FM/FFM. The multiple linear regression analysis showed, in general, that the same variables can be estimated through simple anthropometric measures. This highlights the relevance of the findings of the current study, since simple anthropometric variables can be used to estimate important BIA variables that are related to fat distribution and body composition.

**Abstract:**

Background: Overweight and obesity are conditions associated with sedentary lifestyle and accumulation of abdominal fat, determining increased mortality, favoring chronic diseases, and increasing cardiovascular risk. Although the evaluation of body composition and fat distribution are highly relevant, the high cost of the gold standard techniques limits their wide utilization. Therefore, the aim of this work was to explore the relationships between simple anthropometric measures and BIA variables using multivariate linear regression models to estimate body composition and fat distribution in adults. Methods: In this cross-sectional study, sixty-eight adult individuals (20 males and 48 females) were subjected to bioelectrical impedance analysis (BIA), anthropometric measurements (waist circumference (WC), neck circumference (NC), mid-arm circumference (MAC)), allowing the calculation of conicity index (C-index), fat mass/fat-free mass (FM/FFM) ratios, body mass index (BMI) and body shape index (ABSI). Statistical analyzes were performed with the R program. Nonparametric Statistical tests were applied to compare the characteristics of participants of the groups (normal weight, overweight and obese). For qualitative variables, the Fisher’s exact test was applied, and for quantitative variables, the paired Wilcoxon signed-rank test. To evaluate the linear association between each pair of variables, the *Pearson correlation coefficient* was calculated, and Multivariate linear regression models were adjusted using the stepwise variable selection method, with Akaike Information Criterion (*p* ≤ 0.05). Results: BIA variables with the highest correlations with anthropometric measures were total body water (TBW), body fat percentage (BFP), FM, FFM and FM/FFM. The multiple linear regression analysis showed, in general, that the same variables can be estimated through simple anthropometric measures. Conclusions: The assessment of fat distribution in the body is desirable for the diagnosis and definition of obesity severity. However, the high cost of the instruments (dual energy X-ray absorptiometry, hydrostatic weighing, air displacement plethysmography, computed tomography, magnetic resonance) to assess it, favors the use of BMI in the clinical practice. Nevertheless, BMI does not represent a real fat distribution and body fat percentage. This highlights the relevance of the findings of the current study, since simple anthropometric variables can be used to estimate important BIA variables that are related to fat distribution and body composition.

## 1. Introduction

According to the World Health Organization (WHO), obesity and overweight are defined as an abnormal or excessive accumulation of fat, with negative impact to human health [1]. In 2016, more than 1.9 billion overweight adults were identified, of which 650 million were obese. Overall, about 13% of the world’s adult population were considered obese in the same year. Regarding the global prevalence, the cases of obesity tripled between the years 1975 and 2016. This number is also alarming in children and, in 2019, about 38.2 million children under 5 years identified as overweight or obese. Analyzing the data, the profile of these populations has gradually changed. Before, obesity and overweight were considered a problem in high-income countries, but there was an increasing number of cases in low-income and middle-income countries, especially in urban areas [1]. Nowadays, the prevalence of obesity probably increased due the strategies to avoid contamination during the Coronavirus-19 disease pandemic, as social isolation [2].

The body mass index (BMI) is a simple index used to classify overweight and obesity in adults. It is defined as the body mass in kilograms divided by the height in meters squared (kg/m^2^). It is considered easy to apply, as the values are used for the same sex and for all ages in adults, but the results may not reflect the different degrees of fat in individuals with the same BMI [1,3]. It is important to note that the relationship between BMI and body fat mass is not ideal for accurately estimating the adiposity of the single individual. Body composition is mainly composed of water, fat, proteins, and minerals. Fat is a component that has attracted more attention due to the negative consequences of its accumulation. It is important to distinguish fat from adipose tissue (AT). Most body fat is stored in the AT, but fat can be present in organs, such as the liver and skeletal muscle. The metabolic risk related to the accumulation of fat is strongly dependent on the distribution of fat. Thus, central obesity is a concern in the context of body composition and fat distribution [4], where individuals with greater central obesity are at higher risk of developing cardiometabolic diseases, independently of BMI [5].

The fat distribution occurs mainly in subcutaneous and visceral regions in the body. Studies indicated that the location and distribution of adipose tissue is related to an increased risk of developing diseases than adiposity in general [6,7]. The reduction in muscle mass may favor the development of insulin resistance and cardiovascular diseases [8,9]. Moreover, the total body water (TBW) is generally reduced, and extracellular body water increased in obese women and individuals with sarcopenic obesity [10,11]. This finding would be justified by an edema related to obesity and hormonal responses due to the adipose tissue [11].

Several techniques have been used to assess body composition, such as hydrostatic weighing (densitometry), air displacement plethysmography, dual energy X-ray absorptiometry (DEXA), computed tomography, magnetic resonance, and bioelectrical impedance analysis (BIA), as well anthropometric measures. Among these techniques, anthropometric measures are simple with a low cost associated. However, the best anthropometric measures to assess risks associated with adiposity has not been established so far [12].

Considering the assessment of fat distribution in the body using cheap, easy to use and reliable measures, some studies have proposed the use of BMI, waist circumference (WC), waist-hip ratio (WHR), neck circumference (NC), mid arm circumference (MAC), conicity index (C-index) and A body shape index (ABSI). Some publications have pointed out doubts about the validity of BMI as an indicator of obesity severity, as the BMI does not distinguish between the accumulation of muscle and fat [13]. WC and WHR have been used as complementary measures to BMI to indicate the risk of obesity and, also to predict the risk of mortality more accurately than BMI. Therefore, the use of WC to assess the distribution of abdominal fat is questionable because it is sensitive to body shape (height and body mass), as well as the percentage of fat and its distribution [14].

NC has been used to estimate the accumulation of fat in the upper body segment that may be associated with more lipolytic activity than lower body fat [15]. Other anthropometric parameters have been used to estimate the risk related to obesity and nutritional status, such as MAC, hip circumference (HC) and skinfolds [16]. The C-index has been used to assess obesity and fat distribution. It is based on the idea that (i) the individual accumulates fat around the central region of the trunk, and (ii) the shape of the body resembles a double cone based on ordinary. When the individual has less fat in the central region, the shape of the body resembles a cylinder [17]. Regarding to body composition and fat distribution, changes in fat mass (FM) and free-fat mass (FFM) can be determined by BIA. This model may describe the effects of body composition, as well as the distribution of FM and FFM within the individual variation. In addition, studies also propose the use of an index that presents a relationship between BMI and WC, which is the ABSI [18]. This anthropometric parameter is based on the WC adjusted for height and body mass. ABSI has been used to express excess risk of elevated WC in a convenient and complementary way to BMI and other known risk factors [19].

Since the number of obese people in the world is alarming, it is essential to assess body fat distribution. Although BMI has been used to define obesity, this parameter does not reflect the fat distribution and body fat percentage [20]. Considering the statements about the relevance of the proper evaluation of the body composition with low cost, easy, and effective procedures; the aim of this work was to explore the relationships between simple anthropometric measures and BIA variables using a multivariate linear regression model to estimate body composition and fat distribution in adults.

The hypothesis of this study was that simple anthropometric and BIA measures of body composition and fat distribution would be comparable.

## 2. Methods

### 2.1. Subjects

In this cross-sectional study, 91 individuals were recruited, from May 2018 to October 2019, through a screening performed by the medical staff of *Hospital Universitário Pedro Ernesto (HUPE)*, *Universidade do Estado do Rio de Janeiro* (*UERJ*), Brazil. The analysis were performed in the *Laboratório de Vibrações Mecânicas e Práticas Integrativas* (LAVIMPI), *Policlínica Piquet Carneiro*, *UERJ.* This study was approved by the Research Ethics Committee of the *HUPE*, *UERJ* with the number CAAE 54981315.6.0000.5259, and registered in the *Registro Brasileiro de Ensaios Clínicos* (ReBEC) with the number RBR 2bghmh and UTN: U1111-1181-1177. The principles from the Declaration of Helsinki were followed.

The Strengthening the Reporting of Observational studies in Epidemiology (STROBE) statements were used to report all the different steps of this study [21,22].

The inclusion criteria were: outpatients of both genders, aged over 18 years old. The exclusion criteria were individuals over 60 years old refusing to sign the consent form.

The sample size was calculated considering a regression model with five degrees of freedom for numerator (number of independent variables), with effect size 0.8, significance level 0.01 and power 0.99, the estimated total sample was 50 individuals [23].

### 2.2. Measurements

**Figure 1:**
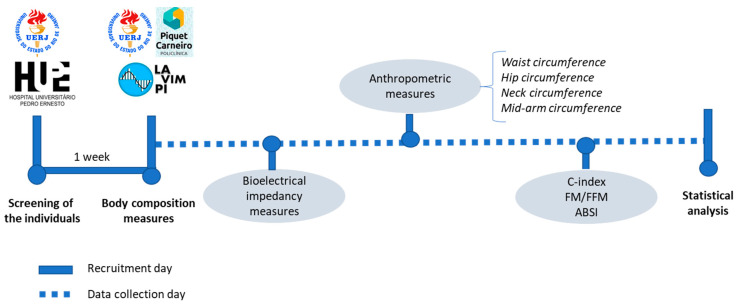
Timeline of the steps of the study. C-index—Onicity index; FM/FFM—Fat mass/fat free mass; ABSI—A body shape index.

Measurements were performed in the individuals according to the presented sequence. A timeline of the steps of the study is presented in the Figure 1.

### 2.3. Bioelectrical Impedance Analysis

TBW (kg), FM (kg) and FFM (kg) were estimated by BIA (In Body 370, BIOSPACE, Korea). The individuals were instructed: to remain at rest for at least 10 min before taking the measurements; to remove metal objects attached to the body (such as rings and earrings); to discontinue the use of diuretic medications for 24 h prior to the measurements; to avoid consumption of food and drink 4 h before the measurements; to avoid practicing physical exercise for 24 h before the measurements; to avoid medicines that promote water retention for 24 h before the measures and to avoid drinking alcohol, coffees and teas for 48 h before the measurements. The subjects were asked to inform about fever in the last days. The measurement procedures were performed according to the manufacturer and other studies [24,25,26,27]. The overweight was considered with BMI ≥ 25 kg/m^2^ and obesity with BMI ≥ 30 kg/m^2^ [1].

### 2.4. Anthropometric Measures: Waist Circumference, Hip Circumference, Neck Circumference, the Mid-Arm Circumference

One trained researcher (anthropometrist accredited by International Society for the Advancement of Kinanthropometry) collected all the anthropometric measures of the individuals. The stature and body mass were measured on a digital balance (MIC 200 PPA, Micheletti, São Paulo, Brazil) and the BMI was calculated as body mass (kg) divided by squared height (m^2^) [1,28].

The measurement of the WC was performed with non-stretchable flexible tapes. The measurement considered the midpoint between the last rib and the iliac crest [1]. The normal WC value of men was considered as ≤94 cm and as ≤80 cm for women [29].

The HC was measured using a non-stretchable tape at the height of the widest point of the hip and was used to calculate the WHR. The individuals were classified as abdominal obesity with WHR values > 0.9 for men and >0.85 for women) [30,31].

The NC was obtained with the individual standing, head in the Frankfurt position, with an inelastic tape measure just below the prominence of the larynx. The mean of three measures was calculated [32]. NC ≥ 35.5 cm for men and ≥32 cm for women was defined as condition of overweight/obesity [33].

To measure the MAC, the individual was instructed to stand upright with the shoulder relaxed and right arm pending, and the instructor stood behind to locate the midpoint between the tip of the olecranon and the acromion. After that, the instructor placed measuring tape around the marked point [34,35].

### 2.5. The C-Index, the FM/FFM Ratios and ABSI

The C-index was calculated from WC (m), body mass (kg) and height (m) using Valdez’s formula: C-index = $\frac{Waist\;Circumference\;\left(m\right)}{0.109\;\sqrt{body\;mass}\;\left(\mathrm{kg}\right)/Height\;\left(m\right)}$.

The C-index ranges from 1.0 (a perfect cylinder) to 1.73 (a perfect double cone), and values increase according to the accumulation of fat in the central region of the body. Thus, the closer to 1.73, the greater the accumulation of abdominal fat [17].

FM and FFM were determined by bioelectrical impedance and the cutoff values were [36]: FM/FFM ratios < 0.40 metabolically healthy obese individuals in whom the increase in FM is minor compared to that in FFM; FM/FFM ratios between 0.40 and 0.80 for obese phenotypes in which FM increases more than FFM, but the FFM is still adequately maintained; FM/FFM ratios > 0.80 for sarcopenic obese phenotypes, in which FM is widely increased and FFM is reduced [37].

The ABSI was calculated according to the following formula WC/(BMI^2/3^.height^1/2^), with WC and height expressed in meters and weight in kilograms [38,39]. The measures have threshold value 0.805 m^11/6^ kg^−2/3^ in women and 0.0828 m^11/6^ kg^−2/3^ in men. The lower value was considered as “lower-ABSI” and the higher was considered as “higher-ABSI” [40].

## 3. Statistical Analysis

Statistical analyzes were performed with the R program [41] and with the R packages: MASS [42], exactRankTests [43], tableone [44], pwr [23]. Nomparametric Statistical tests were applied to compare the characteristics of participants of the groups (normal body mass, overweight and obese individuals). For qualitative variables, expressed in terms of absolute values and percentage, the Fisher’s exact test was applied. For quantitative variables, expressed in terms of median, first and third quartiles, the Kruskal-Wallis test was applied to compare the three groups and for each combination of two groups was applied the Mann-Whitney test. To evaluate the linear association between each pair of variables the *Pearson correlation coefficient* was calculated, also for each pair are presented the scatter plot. Multivariate linear regression models were adjusted using the stepwise variable selection method, with Akaike Information Criterion. The stepwise variable selection method seeks to select the best subset of variables for composing the independent variables in each regression model. Results were considered statistically significant if the *p*-value is under 0.05 (*p* < 0.05).

## 4. Results

**Figure 2:**
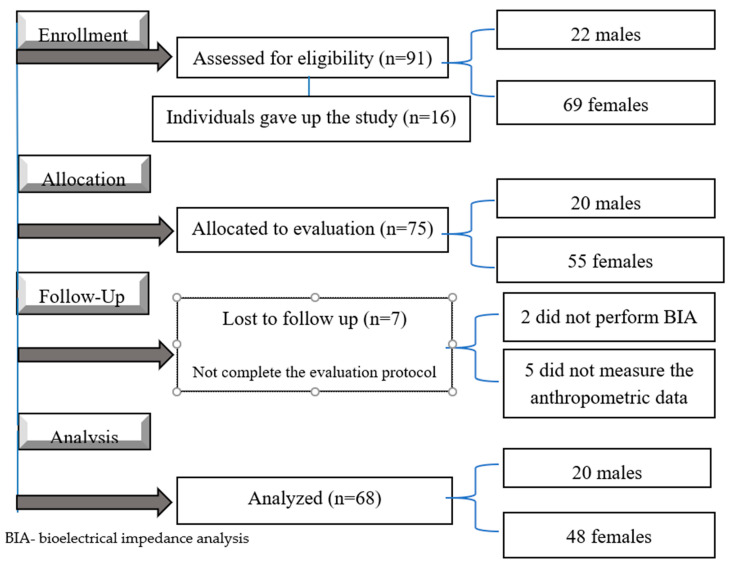
Flow diagram according to the STrengthening the Reporting of OBservational studies in Epidemiology (STROBE) criteria.

The flow diagram of the study is shown in Figure 2. Ninety-one individuals were recruited according to the eligibility criteria, and sixteen individuals declined to participate. Seventy-five individuals were allocated to perform the evaluations and seven of them, performing partially the evaluations, were excluded. Thus, sixty-eight individuals (20 males and 48 females) concluded all the steps of the study.

General characteristics of the participants that concluded all the steps of the study are presented in Table 1. As far as the gender distribution is concerned, the table shows the total number of men and the percentage, while for all other variables are reported the median, first and third quartiles. Considering the three subgroups (normal weight, overweight and obese individuals) no statistical difference was found, except, for the dependent variables involving body composition.

The Pearson’s correlation coefficient ranges from −1 to 1. An absolute value of exactly 1 implies that a linear equation describes the relationship between the variables perfectly. The correlation equal zero indicates that the variables are not correlated. For two variables with positive correlation is expected that if one variable increases the other will also increases, when the signal is negative it is expected that if one variable increases the other will decrease [45].

Table 2 shows the Pearson’s correlation and the associated significant levels (two-tailed *t*-test) for each correlation among anthropometric and bioimpedance variables.

**Figure 3:**
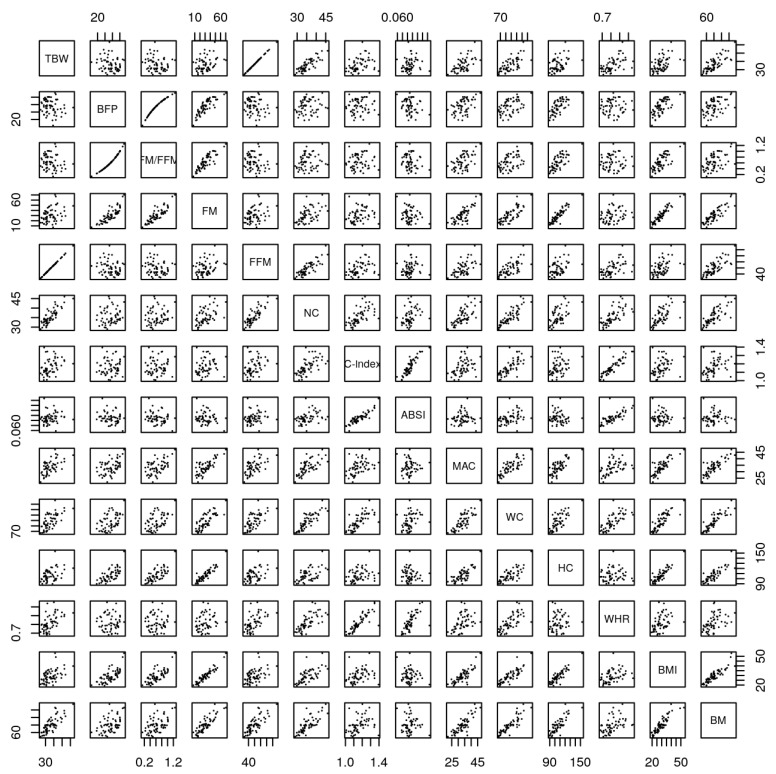
Scatter plot of the variables of the study. TBW—Total body water; FM—Fat mass; BFP—Body fat percentage; FFM—Fat-free mass; BM—Body mass; BMI—Body max index; WHR—Waist-hip ratio; NC—Neck circumference; MAC—Mid-arm circumference; WC—Waist circumference; HC—Hip circumference; C-index—Conicity index; ABSI—A body shape index; FM/FFM—Fat mass/fat-free mass.

Figure 3 shows the scatter plot of anthropometric and BIA variables (TBW, BM; BFP; WHR; NC; MAC; WC; HC; C; ABSI; FM/FFM), presented in Table 2. The following correlations were found: (i) TBW with FM, FFM, NC, C-index, MAC, WC, HC, BMI, BM and WHR, (ii) BFP with FM/FFM, FM, ABSI, MAC, WC, HC, BMI and BM, (iii) FM/FFM with FM, ABSI, MAC, WC, HC, BMI and BM, (iv) FM with FFM, NC, MAC, WC, HC, BMI and BM, (v) FFM with NC, C-index, MAC, WC, HC, BMI, BM and WHR, (vi) NC with C-index, MAC, WC, HC, BMI, BM and WHR, (vii) C-index with ABSI, MAC, WC, BMI, BM and WHR, (viii) ABSI with WC and WHR, (ix) MAC with WC, HC, BMI, BM and WHR, (x) WC with HC, BMI, BM and WHR, (xi) HC with BMI and BM, (xi) BMI with BM and (xii) BM has correlation with WHR.

Table 3 shows the results for Multiple Linear regression analysis, whose coefficients represent the mean change in the response variable for one unit of change in the predictor variable, holding all other predictors constant. Table 3 contains five regressions models, each one has a dependent variable from BIA and a set of independent variables from anthropometric measures.

Related to TBW, the aim of the regression model was to consider the variable TBW as a function of all other anthropometric variables. The stepwise method selected the following independent variables: NC, C-index, ABSI, WHR and BM. The variables NC, ABSI, WHR and BM presented positive coefficients, while the C-index presented negative coefficients.

Related to FM, the aim of the regression model was to model the variable FM as a function of all other anthropometric variables. The stepwise method selected the following independent variables: NC, C-index, ABSI, WHR and BM. The variables C-index and BM presented positive coefficients, while all other variables presented negative coefficients.

Related to BFP, the aim of the regression model was to model the variable BFP as a function of all other anthropometric variables. The stepwise method selected the following independent variables: NC, C-index, ABSI, WHR and BMI. The variable C-index presented positive coefficients, while all other variables presented negative coefficients.

Related to FFM, the aim of the regression model was to model the variable FFM as a function of all other anthropometric variables. The stepwise method selected the following independent variables: NC, C-index, ABSI, WHR and BM. The variable C-index presented a negative coefficient, while all other variables presented positive coefficients.

Related to FM/FFM, the aim of the regression model was to model the variable FM/FFM as a function of all other anthropometric variables. The stepwise method selected the following independent variables: NC, C-index, ABSI and WHR. The variable C-index presented positive coefficients, while all other variables presented negative coefficients.

## 5. Discussion

As it was hypothesized, using a multivariate linear regression model, simple anthropometric measures and BIA measures are comparable to estimate body composition and fat distribution. These findings are highly relevant due to the lack of statistical differences related to the general characteristics of the individuals of the three subgroups (Table 1), with the expected exception of the variables related to body composition.

Several authors have demonstrated the interest in estimating the body composition and fat distribution in adults using anthropometric measures [46,47,48,49], however few studies explored the relation between TBW and obesity. In the current study, TBW (a) correlated with the following anthropometric measures NC, C-index, MAC, WC, HC, BMI, BM and WHR (Table 2 and Figure 3) and (b) with Multiple linear regression analysis, presented positive coefficients to NC, ABSI, WHR and BM, and negative coefficient to C-index (Table 3). These findings are in accordance with those reported by Kuźnar-Kamińska et al., 2017 [50] evaluating the TBW index and anthropometric values such as NC, WC, and abdominal circumference in obese individuals with obstructive sleep apnea. These authors observed an increase in the TBW and anthropometric parameters, but the correlation between these variables was not analyzed. Moreover, the correlation between HC and TBW was considered low, although significant. Mattoo et al., 2020 [51] also suggested that there is a BM decrease related to TBW, which results from a lower % BF. KASHIWAZAKI et al., 1996 [52] studied the prediction equations for total body water and concluded that was necessary to include skinfold measurements together the equation to obtain a valid evaluation for males and females (RAISON et al., 1988).

The current study showed a correlation between the BFP and anthropometric measures, NC, C-index, ABSI, WHR and BM (Table 2 and Figure 3), and with Multiple Linear regression analysis, positive coefficients to C-index, and negative coefficient to NC, ABSI, WHR and BM (Table 3). These findings agree with MATERKO, 2020 [53] that compared the estimate of the BFP by the methods of anthropometry, skinfolds and bioimpedance, demonstrating significant differences between the methods of BIA and skinfolds and between the methods of BIA and anthropometry. Moreover, it is reported that the BIA overestimated the value of BF, thus suggesting that it is not recommended to estimate this variable in obese adult individuals. With respect to linear regression, the BFP showed a significant result when compared to all anthropometric variables that are derived from calculations (C-index, ABSI, BMI and WHR), however it did not present a significant result in relation to NC. Using anthropometric alternatives to estimate BFP is of fundamental importance, as it is widely used to predict the risk of developing diseases such as obesity, diabetes and hypertension, and the reduction of inflammatory factors in the body [54].

The current study showed a correlation between the FM and anthropometric measures C-index, BM (positive correlation), NC, ABSI, WHR and BM (negative correlation) (Table 2 and Figure 3), and with Multiple linear regression analysis, positive coefficients to C-index and BM and negative coefficients to NC, ABSI and WHR (Table 3). These findings agree with a cross-sectional epidemiological study [49,55] describing that all anthropometric indicators were able to diagnose excess FM, as they presented a minimum limit of 95% of the area under the receiver operating characteristic curve (ROC) curve up to 0.50. However, BMI, WHR and WC were more capable of discriminating FM in both sexes compared to the C-Index. Moreover, these results partially corroborate those reported in the current study, where a correlation was found between the FM and C-index. Regarding linear regression, it was possible to identify that FM and WHR were significant, while FM and C-index were not. However, it is reinforced that the use of anthropometric indicators of obesity are relatively simple methods to be evaluated, that can be used as FM discriminators. Among the different methods capable of estimating body composition, they differ in relation to the levels of precision, costs, and difficulty of application. The techniques most used to determine the components of body composition are anthropometry and BIA. Pereira et al. (2015) [56] observed that WC and HC showed a high correlation with BMI and FM [57], these results corroborating those the results of our study. These findings seem to demonstrate that both WC and HC might represent a good option for identifying increases in adiposity. WHR is related to different aspects of body composition and Pereira et al., 2015 showed that WHR had a correlation lower than WC to estimate %BF, but HC showed a similar correlation with WC.

The current study shows that FMM was positively correlated to NC, ABSI, WHR, and BM, while it is negatively correlated to C index. With the regression model, the variable C-index presented a negative coefficient, while NC, C-index, ABSI, WHR and BM presented positive coefficients. Furthermore, FM/FFM ratio was positively correlated to C-index, while negative correlations were observed to NC, ABSI and WHR. Using the regression model, the variable C-index presented positive coefficients, while NC, C-index, ABSI and WHR presented negative coefficients.

Recognizing aspects of body composition is still of great clinical and public health relevance because excess FM and inadequate FFM represent important risk factors for the development of major chronic diseases and mortality, being essential for the development of targeted interventions and effectiveness. However, there is a gap in the literature regarding the relationships between FM/FFM ratio and anthropometric variables [57], and it is suggested that the main limitation of BMI as a predictor of mortality is that it cannot differentiate between FM and FFM.

In the current study a correlation between BMI and FM, FFM and FM/FFM ratio was found, in addition to a correlation of FM/FFM with the anthropometric variables ABSI, WC, MAC and HC. The linear regression analysis showed significance only for the FM/FFM variable with the anthropometric variable WHR, thus suggesting that the aforementioned anthropometric variables are able to predict these BIA variables.

The strength of this study is represented by the observation that simple anthropometric variables can be used to estimate important anthropometric variables that are related to fat distribution and body composition. The major contribution of this study is the proposal to try to “validate” certain anthropometric measurements not related to skinfolds (WC, HC, NC, MAC) with body composition variables monitored by BIA (TBW, FM, FFM).

Considering the limitations of this current work, the anthropometric measurements are compared with a doubly indirect method of body composition that cannot be considered as a reference or gold standard, so the significance of the findings should be taken with caution. Although BIA has actually evolved over the years to include the use of multiple frequencies to improve the accuracy and reliability of body composition estimates, DEXA represents the gold standard test, but this method is expensive and rarely available, thus making its use infeasible in the clinical practice. Although there are no significant differences between the three subgroups analyzed, may be significant differences between men and women. Finally, the small number of the study group and to the disproportion as regards the ratio between the two genders (males were less than females) can be considered as a limitation of this study.

The perspectives of this study are related to development of other studies consid-ering: (i) only analyzes about female individuals (due to the fact that there are few studies involving this specific population); (ii) stratification between female and male individuals; and (iii) analyzes involving individuals with different clinical conditions where the analyze of their body composition can contribute to the management of them.

## 6. Conclusions

Body fat assessment is considered the gold standard for the diagnosis of obesity, however, the high cost of the instruments requested to be used to assess this variable makes the use of BMI the main and most used variable for overweight and obesity. Although all the anthropometric variables considered in the present study correlated with BIA variables, however, when analyzing each variable specifically, discrepancies between the calculations of correlation and linear regression were detected. However, the current study, that presents preliminary results, suggests that linear regression models are useful for estimating dependent variables as a function of independent variables. Nevertheless, further additional studies on huge study populations are requested to confirm these preliminary observations.

## Figures and Tables

**Table 1:** General characteristics of the individuals.

Parameters (Median [IQR]) or (%)	Normal Weight (*n* = 15)	Overweight (*n* = 24)	Obese (*n* = 29)	*p*-Value
Sex = M	3 (20.0%)	7 (29.2%)	10 (34.5%)	0.607
Age (years)	36.00 [33.50, 41.50]	45.50 [40.50, 52.50]	44.00 [37.00, 51.00]	0.078
Body mass (kg)	60.50 [53.20, 63.75] *^,^ᵜ	78.35 [68.78, 80.88] ᵝ	92.80 [79.80, 109.30]	<0.001
Height (m)	1.66 [1.58, 1.71]	1.67 [1.60, 1.70]	1.64 [1.57, 1.73]	0.909
BMI (kg/m^2^)	22.10 [20.70, 23.15] *^,^ᵜ	27.25 [26.45, 28.15] ᵝ	33.80 [32.00, 36.10]	<0.001
NC (cm)	31.57 [30.70, 33.06] *^,^ᵜ	34.15 [33.17, 37.52]	36.75 [34.30, 40.45]	<0.001
MAC (cm)	27.25 [26.61, 29.60] *^,^ᵜ	32.25 [30.28, 33.16] ᵝ	36.20 [34.20, 40.23]	<0.001
WC (cm)	71.12 [69.14, 75.72] *^,^ᵜ	82.50 [79.38, 88.50] ᵝ	100.80 [93.10, 103.38]	<0.001
HC (cm)	94.95 [92.50, 97.22] *^,^ᵜ	103.17 [99.90, 105.50] ᵝ	114.25 [110.09, 121.44]	<0.001
WHR	0.75 [0.72, 0.78] *,ᵜ	0.80 [0.78, 0.86]	0.85 [0.78, 0.93]	<0.001
C-index	1.10 [1.08, 1.11] ᵜ	1.14 [1.10, 1.18]	1.20 [1.14, 1.25]	0.002
ABSI	0.07 [0.07, 0.07]	0.07 [0.07, 0.07]	0.07 [0.07, 0.07]	0.953
FM/FFM	0.38 [0.31, 0.45] *,ᵜ	0.60 [0.49, 0.64] ᵝ	0.82 [0.65, 0.92]	<0.001
TBW (kg)	30.90 [28.20, 33.15] ᵜ	35.75 [30.85, 39.68]	38.00 [32.40, 44.40]	0.007
Proteins (kg)	8.20 [7.60, 8.90] ᵜ	9.50 [8.28, 10.62]	10.40 [8.70, 12.10]	0.006
Minerals (kg)	3.10 [2.76, 3.33] ᵜ	3.51 [2.99, 3.75]	3.53 [3.04, 4.31]	0.024
FM (kg)	16.10 [13.80, 18.45] *	26.25 [24.23, 30.40] ᵝ	39.10 [34.60, 47.90]	<0.001
Lean mass (kg)	39.70 [36.30, 42.60] ᵜ	45.85 [39.70, 51.08]	49.00 [41.60, 57.20]	0.007
FFM (kg)	42.20 [38.55, 45.35] ᵜ	48.70 [42.25, 54.20]	51.90 [44.20, 60.70]	0.008
BFP (%)	27.80 [23.60, 30.85] *	37.60 [32.85, 39.05] ᵝ	45.00 [39.40, 48.00]	<0.001

IQR—Nterquartile range; M—Male; BMI—Body max index; NC—Neck circumference; MAC—Mid-arm circumference; WC—Waist circumference; HC—Hip circumference; WHR—Waist-hip ratio; C-index—Conicity index; ABSI—Body shape index [WC/(BMI^2/3^ *Height^1/2^)]; FM/FFM—Fat mass/fat-free mass; TBW—Total body water; FM—Fat mass; FFM—Fat-free mass; BFP—Body fat percentage; * when normal are different of overweight; ᵜ difference between normal and obese; ᵝ difference between overweight and obese *p* ˂ 0.05.

**Table 2:** Pearson’s correlations of the studied variables.

	TBW	BFP	FM/FFM	FM	FFM	NC	C-Index	ABSI	MAC	WC	HC	BMI	BM	WHR
**TBW**	-	−0.18	−0.17	0.33	1.00	0.84	0.36	0.13	0.59	0.66	0.35	0.48	0.78	0.56
**BFP**	ns	-	0.98	0.84	−0.18	0.12	0.07	−0.27	0.47	0.48	0.45	0.70	0.46	−0.03
**FM/FFM**	ns	c	-	0.87	−0.17	0.14	0.08	−0.27	0.51	0.50	0.45	0.74	0.49	−0.02
**FM**	a	c	c	-	0.32	0.51	0.23	−0.21	0.77	0.78	0.63	0.94	0.85	0.22
**FFM**	c	ns	ns	a	-	0.83	0.35	0.12	0.59	0.66	0.35	0.47	0.77	0.56
**NC**	c	ns	ns	c	c	-	0.62	0.33	0.68	0.85	0.38	0.64	0.78	0.70
**C-index**	a	ns	ns	ns	a	c	-	0.89	0.39	0.73	0.17	0.29	0.34	0.84
**ABSI**	ns	a	a	ns	ns	a	c	-	0.03	0.35	−0.10	−0.17	−0.07	0.70
**MAC**	c	b	c	c	c	c	a	ns	-	0.79	0.60	0.82	0.83	0.42
**WC**	c	b	c	c	c	c	c	a	c	-	0.52	0.84	0.87	0.72
**HC**	a	b	b	c	a	a	ns	ns	c	c	-	0.63	0.61	0.05
**BMI**	c	c	c	c	c	c	a	ns	c	c	c	-	0.89	0.37
**BM**	c	c	c	c	c	c	a	ns	c	c	c	c	-	0.46
**WHR**	c	ns	ns	ns	c	c	c	c	c	c	ns	ns	b	-

TBW—Total body water; BM—Body mass; BFP—Body fat percentage; WHR—Waist-hip ratio; NC—Neck circumference; MAC—Mid-arm circumference; WC—Waist circumference; HC—Hip circumference; C-index—Conicity index; ABSI—A body shape index; FM/FFM—Fat mass/fat-free mass. (a = *p* ≤ 0.05; b = *p* ≤ 0.001; c = *p* ≤ 0.0001; ns = *p* > 0.05).

**Table 3:** Multiple Linear regression analysis.

Multiple Linear Regression, Dependent Variable TBW			Multiple Linear Regression, Dependent Variable FM		
Variables	Coefficients	*p*-value	Variables	Coefficients	*p*-value
(Intercept)	−22.10	0.00000546	(Intercept)	29.50	0.00000617
NC	0.99	0.000000126	NC	−1.33	0.000000128
‘C-index’	−198.51	2.00E-16	‘C-index’	275.00	2.00E-16
ABSI	2660.69	5.13E-15	ABSI	−3685.00	1.71E-15
WHR	29.25	0.0000336	WHR	−40.20	0.0000237
BM	0.47	1.28E-15	BM	0.35	5.49E-08
Adjusted R-squared: 0.9322			Adjusted R-squared: 0.9506		
Multiple linear regression, dependent variable BFP			Multiple linear regression, dependent variable FFM		
Variables	Coefficients	*p*-value	Variables	Coefficients	*p*-value
(Intercept)	126.35	4.07E-09	(Intercept)	−29.54	0.00000602
NC	−1.51	3.39E-08	NC	1.33	0.000000128
‘C-index’	656.62	0.00000034	‘C-index’	−275.15	2.00E-16
ABSI	−9468.26	0.00000063	ABSI	3687.07	1.66E-15
WHR	−62.14	0.000143	WHR	40.24	0.0000233
BMI	−2.13	0.003251	BM	0.65	4.13E-16
Adjusted R-squared: 0.839			Adjusted R-squared: 0.934		
Multiple linear regression, dependent variable FM/FFM					
Variables	Coefficients	*p*-value			
(Intercept)	1.58	1.53 × 10^−11^			
NC	−0.05	8.33 × 10^−12^			
‘C-index’	8.53	2.00 × 10^−16^			
ABSI	−117.00	2.00 × 10^−16^			
WHR	−0.89	0.00205			
Adjusted R-squared: 0.8577					

TBW—Total body water; BM—Body mass; BFP—Body fat percentage; NC—Neck circumference; HC—Hip circumference; C-index—Conicity index; ABSI—A body shape index; BMI—Body mass index; MAC—Mid-arm circumference; WC—Waist circumference; WHR—Waist hip circumference.

## Data Availability

The datasets used and/or analyzed during the current study are available from the corresponding author on reasonable request.
